# Simultaneously achieving high capacity storage and multilevel anti-counterfeiting using electrochromic and electrofluorochromic dual-functional AIE polymers†

**DOI:** 10.1039/d1sc00722j

**Published:** 2021-04-12

**Authors:** Lin Lu, Kaojin Wang, Haozhong Wu, Anjun Qin, Ben Zhong Tang

**Affiliations:** State Key Laboratory of Luminescent Materials and Devices, Guangdong Provincial Key Laboratory of Luminescence from Molecular Aggregates, AIE Institute, Center for Aggregation-Induced Emission, South China University of Technology Guangzhou 510640 China msqinaj@scut.edu.cn; Shenzhen Institute of Aggregate Science and Technology, School of Science and Engineering, The Chinese University of Hong Kong Shenzhen Guangdong 518172 China

## Abstract

With the advent of the big data era, information storage and security are becoming increasingly important. However, high capacity information storage and multilevel anti-counterfeiting are typically difficult to achieve simultaneously. To address this challenge, herein, two electrochromic and electrofluorochromic dual-functional polymers with aggregation-induced emission (AIE) characteristics were rationally designed and facilely prepared. Upon applying voltages, the absorption and fluorescence spectra of the AIE polymers can undergo reversible changes, accompanied by variation of their color and emission. By utilizing the controllable characteristics of the polymers, dual-mode display devices were fabricated *via* a simple spraying technique. More interestingly, a four-dimensional color code device was constructed by adding color change multiplexing to the two-dimensional space, thereby achieving high capacity information storage. Moreover, the color code device can also be applied in the multilevel anti-counterfeiting area. The encrypted information can be dynamically converted under different voltages. Thus, the AIE polymers show great promise for applications in multidimensional information storage and dynamic anti-counterfeiting, and the design strategy may provide a new avenue for advanced information storage and high security technology.

## Abstract 2

By using electrical stimuli-responsive AIE polymers, dual-mode display devices, multidimensional information storage and anti-counterfeiting devices were constructed.

## Graphical abstract

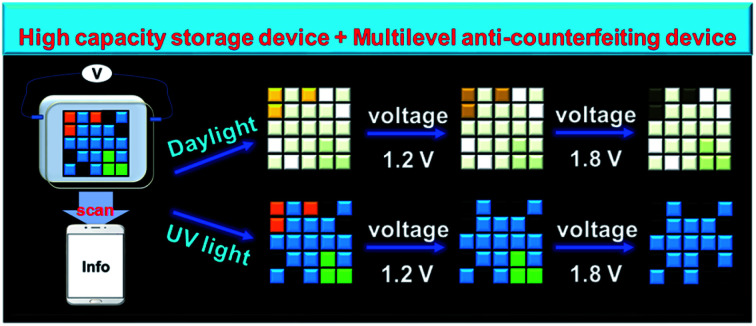

## Introduction

With the development of the big data era, global information is increasing explosively, raising huge challenge to information storage capacity.^1–3^ Meanwhile, counterfeiting threatens the information security of both individuals and society.^4,5^ Therefore, it is highly desirable to realize both high capacity information storage and multilevel anti-counterfeiting in the same media. To increase the information storage capacity within a spatially limited volume, information multiplexing shows distinct advantages by expanding the two-dimensional (2D) physical space to a multidimensional one.^6–8^ A few breakthroughs have been reported by addition of distinguishable new dimensions to 2D space, such as wavelength,^9,10^ intensity,^11,12^ polarization,^13^ and time.^14–16^ In terms of anti-counterfeiting, stimuli-responsive materials have been widely explored to address the counterfeiting challenge because of their unique response properties. Smart materials exhibit obvious changes under stimuli, such as light, electricity, mechanical force, heating, pH, and their combinations.^17–25^ Specifically, some remarkable reports have achieved multiple transformations of output in response to the stimulus input, rather than just output in a static form, which could further realize multilevel security.^26–29^ Consequently, to simultaneously satisfy the requirements of high capacity information storage and anti-counterfeiting, a suitable medium needs to meet at least two criteria: (1) expansion of 2D physical space to multidimensional space, and (2) multilevel transformations under stimulation. Apparently, it is a great challenge to explore appropriate systems possessing the advantages of both high capacity storage and multilevel security.

Among these stimuli-responsive materials, electrically responsive ones, such as electrochromic and electrofluorochromic materials, have attracted tremendous attention owing to the reversible changes in their optical properties, including color, transmittance, and fluorescence, caused by redox-driven electric potentials.^30–35^ Notably, to address the low fluorescence contrast in the solid state, a few electrochromic and electrofluorochromic dual-functional materials have been developed by introducing aggregation-induced emission (AIE) units.^36–38^ Taking advantage of the controllable stimulus change, these materials have been exploited in multicolor displays, smart windows, E-paper, sunglasses, *etc.*^39–44^ However, less attention has been paid to anti-counterfeiting applications,^45–49^ and the actual demand for high capacity storage and multilevel anti-counterfeiting cannot be realized by the reported technology. In principle, by combining the characteristics of controllable color and fluorescence of electrochromic and electrofluorochromic dual-functional materials, it may be possible to simultaneously realize information multiplexing storage and dynamic anti-counterfeiting.

**Fig. 1:**
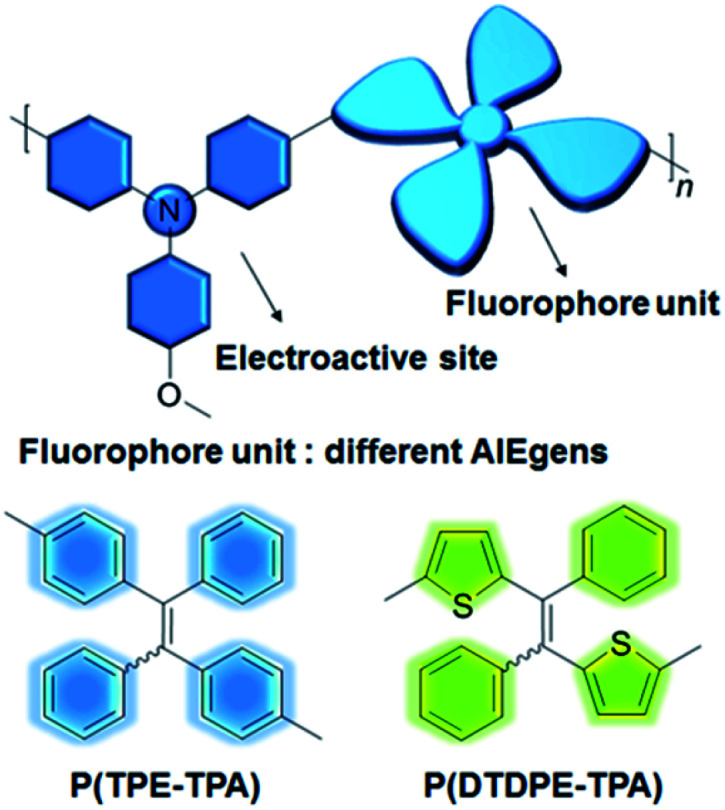
Design principle for electrical stimulus-responsive polymers with different AIE units.

Herein, we put forward a simple but effective strategy for constructing information storage and multilevel anti-counterfeiting devices using electrochromic and electrofluorochromic dual-functional AIE polymers. As shown in Fig. 1, 4-methoxytriphenylamine (TPA-OMe) was chosen as the electroactive modulator since it is regarded as a shining star in the electrochromic realm owing to its high electrochemical stability and low oxidation potential. To improve the fluorescence intensity of the material in the solid state, two different AIE units, tetraphenylethylene (TPE) and dithiophenyldiphenylethylene (DTDPE), were introduced into the polymers. By changing the structures of the polymer backbones, the absorption, fluorescence, and electrical stimulation performance of the resultant P(TPE-TPA) and P(DTDPE-TPA) can be well controlled on demand. Under stimulation by different voltages, the color and fluorescence intensity of the two polymers changed obviously. Thanks to their good solubility, the polymers can be used to construct differently patterned display and transformation devices by a spraying technique. More excitingly, a four-dimensional color code device was fabricated, and dynamic transformation could be realized under electrical stimulation, showing potential in multidimensional information storage and multilevel anti-counterfeiting applications. To the best of our knowledge, this is the first example of four-dimensional color code device technology based on electrical stimulus-responsive materials, which may broaden the applications of these materials in advanced storage and security areas.

## Results and discussion

### Synthesis and characterization

The synthetic routes to P(TPE-TPA) and P(DTDPE-TPA) are outlined in Scheme S1 (ESI†), and the synthesis details are provided in the ESI. The TPA-OMe monomer was synthesized according to the reported procedure.^50^ The TPE-Br and DTDPE-Br co-monomers were synthesized through McMurry coupling. P(TPE-TPA) and P(DTDPE-TPA) were then readily obtained through Suzuki polycoupling. To facilitate the application, we also designed and synthesized a third polymer, P(TPE-NB), which shows no response to electrical stimulation (Scheme S2, ESI†).

All the monomers and polymers were fully characterized by ^1^H and ^13^C NMR spectroscopy and satisfactory results were obtained (Fig. S1–S15, ESI†). P(TPE-TPA) and P(DTDPE-TPA) are soluble in common organic solvents, such as tetrahydrofuran (THF) and chloroform, which facilitates the fabrication of thin films. The decomposition temperatures (*T*_d_, 5% weight loss) of P(TPE-TPA) and P(DTDPE-TPA) were measured to be as high as 451 and 460 °C by thermogravimetric analysis, and their glass transition temperatures were found to be 223 and 230 °C by differential scanning calorimetry, respectively (Fig. S16, ESI†). After confirming their excellent thermal properties, the electrochemical properties of P(TPE-TPA) and P(DTDPE-TPA) were measured by cyclic voltammetry. As shown in Fig. S17 (ESI†), the polymer films show relatively low onset oxidation voltages (*E*_onset_), which were observed at 0.62 V for P(TPE-TPA) and 0.43 V for P(DTDPE-TPA). It is worth noting that *E*_onset_ of P(DTDPE-TPA) is lower than that of P(TPE-TPA) due to the stronger electron donating ability of thiophene groups than phenyl groups in the polymer main chains.

### Photophysical properties

The UV-vis absorption and photoluminescence (PL) spectra were recorded to analyze the photophysical properties of P(TPE-TPA) and P(DTDPE-TPA), and the data are summarized in Table S1 (ESI†). As shown in Fig. S18 (ESI†), P(TPE-TPA) shows a maximum absorption peak at 372 nm in THF solution, whereas, after incorporation of the electron donating thiophene groups in P(DTDPE-TPA), the peak was red-shifted to 441 nm. Upon excitation at the maximum absorption wavelength, P(TPE-TPA) exhibits green emission with a PL peak at 513 nm in dilute THF solution, and shows an absolute PL quantum yield (*Φ*_F_) of 2.2%. Because of its more planar conformation and stronger π-conjugation than P(TPE-TPA), P(DTDPE-TPA) shows a red-shifted PL peak at 585 nm. However, it displays a *Φ*_F_ value of only 0.7%, which could be ascribed to the quenching effect of the sulfur atoms in the thiophene rings. In contrast to the faint emission in THF solution, bright emission of P(TPE-TPA) and P(DTDPE-TPA) with *Φ*_F_ values of 52.9 and 15.6%, respectively, was recorded in the film state, indicative of typical AIE characteristics.

**Fig. 2:**
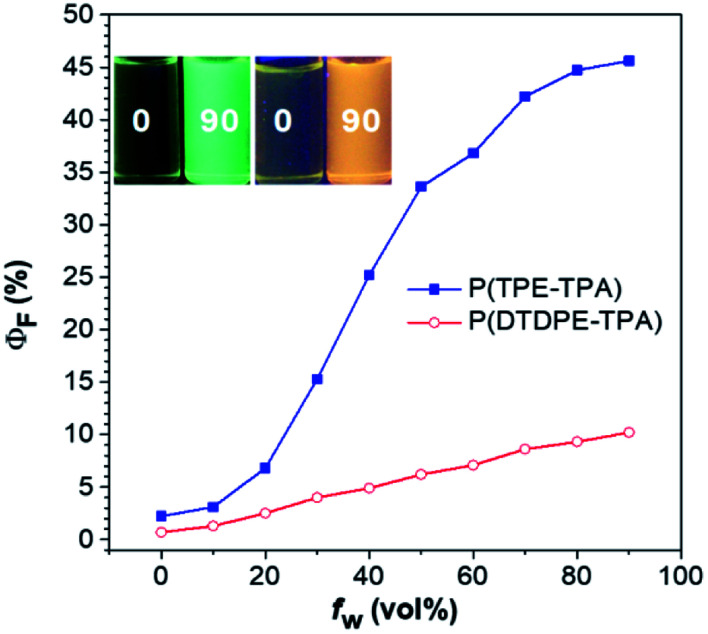
Plots of absolute PL quantum yield (*Φ*_F_) values *versus f*_w_ in THF/water mixtures. Inset: photographs of P(TPE-TPA) (left) and P(DTDPE-TPA) (right) in THF and THF/water mixtures with a *f*_w_ of 90%.

To further investigate the AIE activity, the emission behavior of the two polymers was analyzed in THF/water mixtures with different water fractions (*f*_w_), where water is a poor solvent for them. As displayed in Fig. 2, the *Φ*_F_ values of the two polymers are significantly enhanced with an increase in *f*_w_, and the highest values were recorded in THF/water mixtures with 90% *f*_w_, and are 21- and 15-fold higher than those in THF solutions for P(TPE-TPA) and P(DTDPE-TPA), respectively. The PL spectra of P(TPE-TPA) and P(DTDPE-TPA) in THF/water mixtures with different *f*_w_ further confirm their AIE behavior (Fig. S19, ESI†). The AIE properties of P(TPE-TPA) and P(DTDPE-TPA) can also be vividly seen from photos of THF solutions and THF/water mixtures with *f*_w_ of 90% taken under UV irradiation. The AIE activity of these two polymers could be well explained by restriction of intramolecular motion.^51^

### Electrochromic and electrofluorochromic performance

**Fig. 3:**
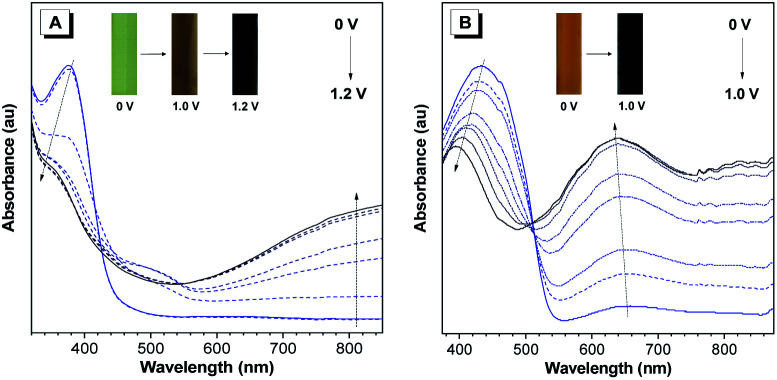
UV spectra of (A) P(TPE-TPA) and (B) P(DTDPE-TPA) thin film electrodes in 0.1 M tetrabutylammonium perchlorate (TBAP)/CH_3_CN solutions under different voltages. Inset: photographs of the polymer films at different voltages.

The electrochromic properties of P(TPE-TPA) and P(DTDPE-TPA) in the film state were studied on an electrochemical workstation combined with UV-vis spectroscopy. As shown in Fig. 3A, when the applied potential increased from 0.0 to 1.2 V, the absorption band below 440 nm gradually decreased while a new absorption band at longer wavelength (500–800 nm) emerged and progressively increased, which could be attributed to the formation of cation radicals in the polymer main chains during the electrooxidation process.^52,53^ The color of the P(TPE-TPA) film changed from green (0.0 V) to brown (1.0 V) and finally to black (1.2 V). Similar modulated behavior of the P(DTDPE-TPA) film was observed after increasing the potential from 0.0 to 1.0 V (Fig. 3B). The neutral absorption peak centered at 435 nm gradually decreased while a new absorption peak at around 633 nm grew, accompanied by a color change from orange to black.

Afterwards, the response time and switching stability of the polymers were characterized by monitoring their absorption changes after applying sequential square wave potentials. The switching times of the films required to reach 90% of their maximum absorption changes were calculated. As shown in Fig. S20 (ESI†), the coloring/blenching response times of P(TPE-TPA) and P(DTDPE-TPA) are calculated to be 2.2/4.0 s for P(TPE-TPA) and 2.1/3.8 s for P(DTDPE-TPA), which are comparable with previous work.^54–56^ These data suggest that they could display obvious color changes in a short time through electrical stimulus, which is conducive to anti-counterfeiting applications. Notably, as displayed in Fig. S21 (ESI†), the polymers maintain good recoverability even after 50 cycles.

In addition, the coloration efficiency (CE), which is also one of the key parameters for electrochromic materials, was calculated according to the equation CE = ΔOD/*Q*, representing the change in optical density (ΔOD) per unit of injected or ejected charge density (*Q*) at a certain wavelength.^57^ For P(TPE-TPA), ΔOD at 373 nm is 0.38, and *Q* is 3.78 mC cm^−2^, whereas, for P(DTDPE-TPA), ΔOD at 431 nm is 0.43, and *Q* is 3.55 mC cm^−2^ (Fig. S22, ESI†). Accordingly, P(TPE-TPA) and P(DTDPE-TPA) show CE values of 101 and 121 cm^2^ C^−1^, respectively, which are comparable with reported systems.^36,58^ Compared with P(TPE-TPA), the more conjugated P(DTDPE-TPA) shows a higher CE, indicating larger optical modulation with a smaller charge effect.

**Fig. 4:**
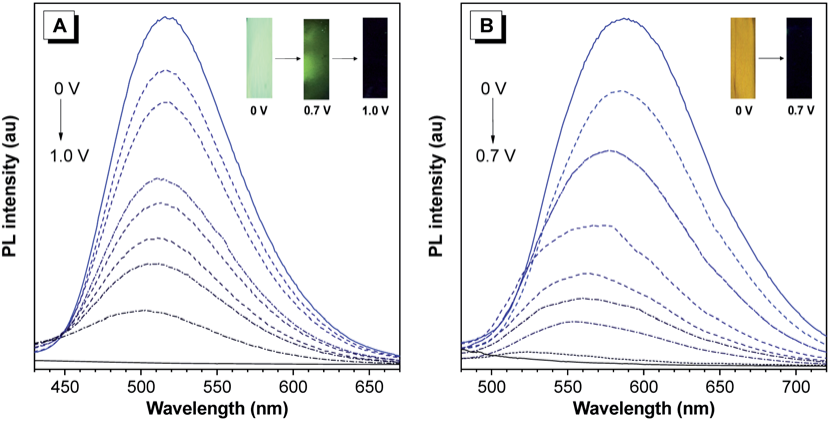
PL spectra of (A) P(TPE-TPA) and (B) P(DTDPE-TPA) thin film electrodes in 0.1 M TBAP/CH_3_CN solution under different voltages. Inset: photographs of the two polymer films at different voltages under UV light.

Thanks to their AIE features, we then studied the fluorescence change in films of P(TPE-TPA) and P(DTDPE-TPA) by applying different positive potentials. As shown in Fig. 4A, the strong green fluorescence of the P(TPE-TPA) film was quenched to dark green at an applied potential of 0.7 V, then almost vanished at 1.0 V. Similarly, the PL intensity of the P(DTDPE-TPA) film decreased upon increasing the potential from 0 to 0.7 V (Fig. 4B). The quenching behavior was attributed to the generation of cationic radicals, which act as an effective fluorescence quencher due to the large spectral overlap between the absorption and the emission in the oxidation process.^53^ It is worth noting that the fluorescence could be recovered by applying a reverse voltage, and this off/on process could be cycled several times (Fig. S23, ESI†). Based on the above experiments, the fluorescence on/off contrast ratio was calculated, and the value is 282 for P(TPE-TPA) and 146 for P(DTDPE-TPA), which are higher than those in most reports.^59–61^ Moreover, the fluorescence off/on response times of P(TPE-TPA) and P(DTDPE-TPA) were estimated at 90% of the full switching of their film states (Fig. S24, ESI†), and were deduced to be 1.4/38 and 0.8/36 s respectively. The shorter response time of P(DTDPE-TPA) than that of P(TPE-TPA) could be attributed to the thiophene units in the former that make the polymer more conjugated, thereby enhancing the intramolecular charge transport ability.^53^ The relatively long recovery time of the fluorescence might be due to its sensitivity to the surrounding environment, which takes time to be restored.^62^ Interestingly, the voltages required for complete fluorescence quenching of the two polymers are different, which facilitates the following electrically stimulated applications.

### Dual-mode display device

**Fig. 5:**
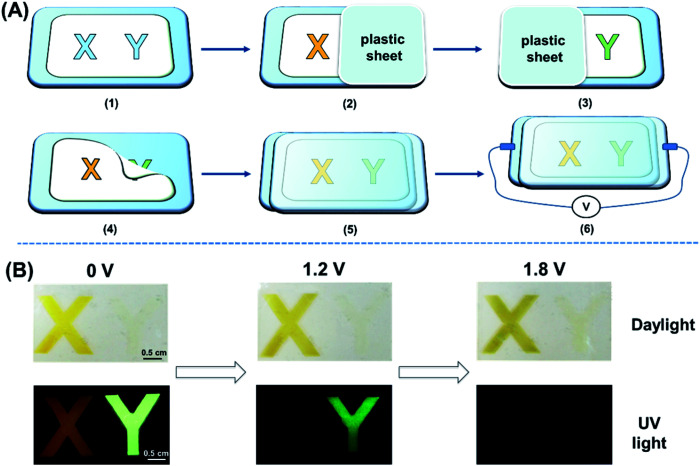
(A) Schematic illustration of the fabrication procedure of the device: (1) stick the mold on the ITO glass; (2) block the “Y” area with a plastic sheet and spray P(DTDPE-TPA) in the “X” area; (3) block the “X” area with a plastic sheet and spray P(TPE-TPA) in the “Y” area; (4) remove the pattern mold; (5) fill with gel electrolyte and cover with another ITO glass; and (6) construct the device and apply different voltages. (B) Photographs showing the changes in the “X” and “Y” patterns at different voltages under daylight and UV light. The size of the ITO substrate is 4.5 × 5.5 cm.

Encouraged by the unique performance of the polymers under electrical stimulus, we first constructed a dual-mode display device with the configuration of ITO glass/polymer/gel electrolyte/ITO glass, and the device fabrication details are described in Fig. 5 and the ESI.† A polyvinyl chloride (PVC) mold with a thickness of 0.5 mm and “X” and “Y” patterns with an area of 1.5 × 1.5 cm was put on a clean ITO substrate. Afterwards, P(DTDPE-TPA) in chloroform with a concentration of 5 mg mL^−1^ was sprayed in the “X” area thrice while blocking “Y” with a plastic plate to keep it clean. By repeating the same process and using P(TPE-TPA) as the polymer, the “Y” area was also filled. To prevent leakage problems after removing the mold and covering with another ITO plate, a gel electrolyte with the composition of acetonitrile/tetrabutylammonium perchlorate/propylene carbonate/polymethyl methacrylate (70 : 3 : 20 : 7, wt%, ESI†) was employed to construct the device.

Under daylight, the colors of the “X” and “Y” patterns changed significantly with increasing voltage (Fig. 5B and Movie S1, ESI†). Upon UV irradiation, the orange emission of “X” and the green emission of “Y” can be seen at 0 V. The “X” rapidly experienced fluorescence quenching when the positive voltage was increased to 1.2 V, and only the green emissive “Y” could be observed due to the difference in voltage response. Upon further increasing the voltage to 1.8 V, the emission of both the “X” and “Y” patterns completely disappeared (Fig. 5B). Therefore, the device can be applied in dual-mode displays under daylight and UV light. Notably, compared with fluorescence experiments in liquid electrolyte, the device requires a higher voltage to quench the fluorescence due to the slow ion transfer rate of the gel electrode and the increased redox potential in the device.^53,62^

To prove the universality of this display approach, we also constructed a numerical device with an “8” pattern using the above procedure (Fig. S25 and Movie S2, ESI†), in which the left vertical lines and the three horizontal lines were composed of P(DTDPE-TPA) and P(TPE-TPA), respectively. To increase the diversity of color, the electrically inactive and blue emissive P(TPE-NB) bearing TPE side chains was introduced into the device as the right vertical lines. The numerical device can display three different numbers, “8”, “3”, and “1”, at 0, 1.2, and 1.8 V, respectively. These results demonstrate that the device design strategy and the simple spraying method can realize dual-mode displays and dynamic conversion of the patterns.

### High capacity storage device

Inspired by the above color display devices, we speculated that the realization of high capacity information storage is possible by constructing a four-dimensional color code device. To confirm this, a device was prepared by employing the aforementioned procedure and configuration, and P(TPE-TPA), P(DTDPE-TPA), and P(TPE-NB) were selectively sprayed into the designated grids on an ITO substrate according to the specified combination.

**Fig. 6:**
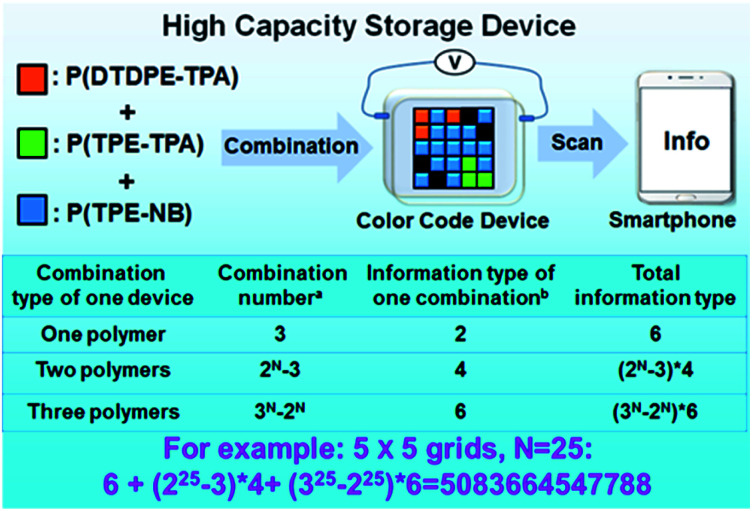
Schematic illustration of the high capacity storage device. The combination number is based on the mathematical arrangement; the total information types include the color and fluorescence, and the information content is transformed by electrical stimuli under daylight and UV light, respectively. A device with 5 × 5 grids (*N* = 25) can store over five trillion information types.

As depicted in Fig. 6, the designed device increases the color change multiplexing on the basis of the 2D physical space and expands it to multidimensional space, thereby increasing the information density per unit area. The arrangement of the three polymers in the grids and the total information obtained can be calculated by mathematical combination. Theoretically, by scanning the programmed color code, the information could be easily read by a smartphone.

When only one polymer is sprayed onto ITO, there are three combinations in the grids that can give two different kinds of information under daylight and UV light. In the case of two polymers, there are two options in each grid. At the same time, the color of the device will change under electrical stimulus; accordingly four different messages in one device will be stored under daylight and UV light. In the presence of three polymers, there are three choices in each grid. And the information changes twice under electrical stimulus, so six different kinds of information in one device can be obtained. If a device with 5 × 5 grids is fabricated, over five trillion total stored information types could be achieved, which readily overcomes the capacity limit dictated by 2D space. If the number of grids in the device further increases, the obtained information number could be almost endless, displaying ultra-high capacity storage.

### Multilevel anti-counterfeiting device

**Fig. 7:**
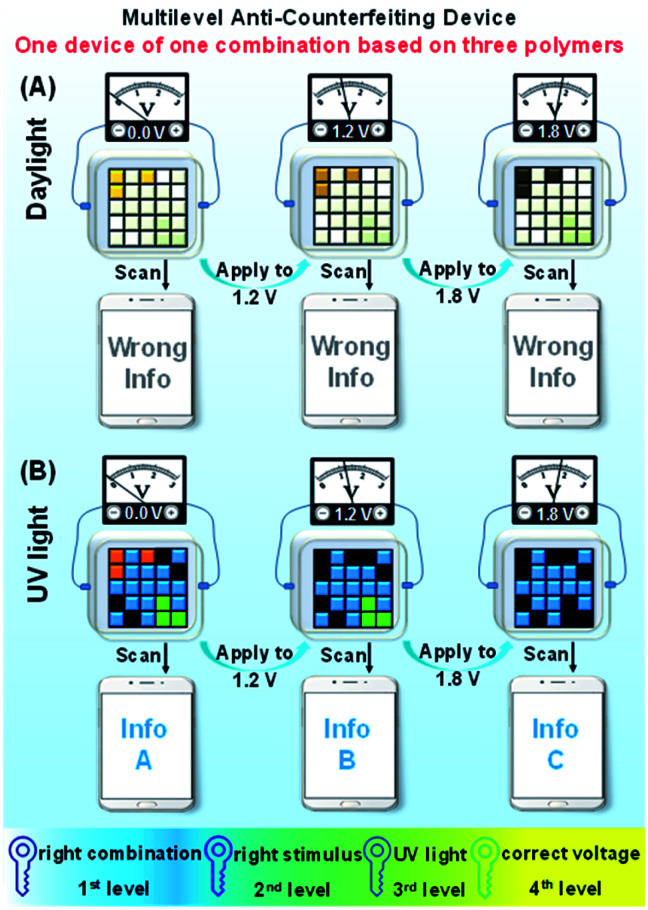
Schematic illustration of a multilevel anti-counterfeiting device using a combination of P(TPE-TPA), P(DTDPE-TPA), and P(TPE-NB) under (A) daylight and (B) UV light.

Thanks to the dynamical control of the electrochromic and electrofluorochromic properties, the color code device also has potential in information anti-counterfeiting applications. As schematically demonstrated in Fig. 7, under different potentials, the color and fluorescence of certain areas in the color code device would be changed, and accordingly dynamic manipulation would be achieved. The information read through a smartphone under daylight was defined as the wrong message, whereas the information read under UV light was defined as the right one. Hence, true information can only be read by a smartphone upon UV irradiation. The security of information encrypted through this new strategy is extremely high. The counterfeiter must identify all (not just some) of the following security measures: (1) the right combination of the three polymers; (2) the right stimulus (electricity); (3) the correct observation conditions (UV light); (4) the appropriate voltage value to obtain the information. Consequently, only an authorized individual who knows the correct decoding rules can access the confidential information, greatly improving the security level of the information.

**Fig. 8:**
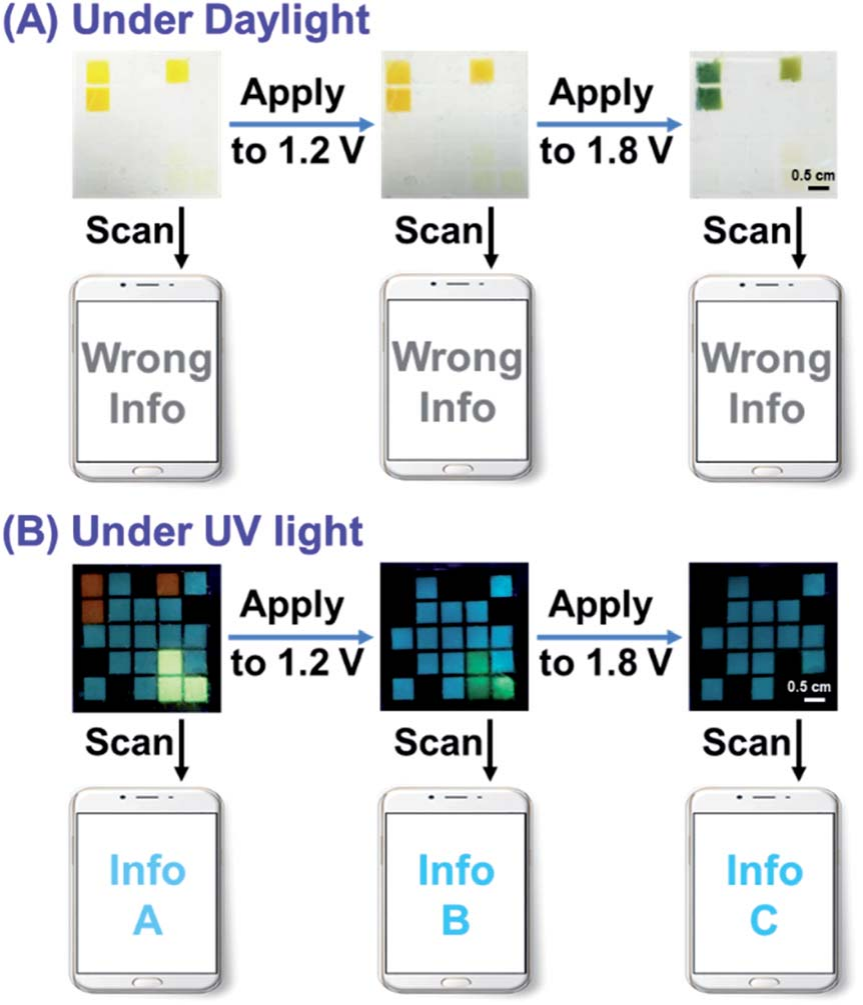
The color code devices based on the combination of P(TPE-TPA), P(DTDPE-TPA), and P(TPE-NB) at different voltages under (A) daylight and (B) UV light. The size of the ITO substrate is 4.8 × 4.8 cm.

Based on the above idea, we fabricated color code devices. First, a device using a combination of P(TPE-TPA) and P(DTDPE-TPA) was successfully constructed (Fig. S26 and Movie S3, ESI†), which confirmed our analysis. We thus fabricated color code devices using a combination of P(TPE-TPA), P(DTDPE-TPA), and P(TPE-NB) (Fig. 8 and Movie S4, ESI†). The correct encoded information in these devices could only be read under UV light, while the information could be recognized as a wrong message by smartphone scanning under daylight.

In the initial state, the blue, orange, and green fluorescence of the color code device was clearly seen, and the specific information (Info A) could be read from a smartphone when the device was irradiated with UV light. After increasing the voltage to 1.2 V, the orange fluorescence of P(DTDPE-TPA) was quenched, and only the blue and green fluorescence colors could be identified by smartphone, thus obtaining other information (Info B). Further increasing the voltage to 1.8 V led to P(TPE-TPA) becoming non-emissive, and the color code device only emitted blue light, which could be read as Info C. Notably, upon modulating the voltage, Info A, Info B, and Info C could be dynamically transformed. These device results indicate that different true information was decrypted upon UV irradiation at various voltages. Hence, only an authorized individual knowing the correct encryption algorithm has the opportunity to obtain the specific information, which guarantees information security.

## Conclusions

We rationally designed and facilely prepared two electrochromic and electrofluorochromic dual-functional AIE polymers, P(TPE-TPA) and P(DTDPE-TPA), based on which high capacity storage and multilevel anti-counterfeiting were simultaneously realized. By using a simple spraying method, dual-mode display devices with different patterns (“X”, “Y” and “8”) were successfully constructed. High capacity information storage was achieved by spatial arrangements, color (visible or emission), and electrical inputs, which overcame the limitations of 2D space. Taking advantage of the controllable change feature, color code devices can also be applied in the multilevel anti-counterfeiting area, in which the dynamic transformation of Info A, Info B, and Info C was realized. Thus, this work provides new insight into the exploration of electrochromic and electrofluorochromic dual-functional AIE polymers in information storage and dynamic security applications, which might open up new avenues for electrical stimulus responsive materials.

## Author contributions

L. Lu and A. Qin designed and planned the experiments. L. Lu prepared samples and carried out tests. L. Lu, H. Wu, A. Qin and B. Z. Tang analyzed data and discussed the results. L. Lu, K. Wang, A. Qin and B. Z. Tang contributed to writing and editing the paper.

## Conflicts of interest

There are no conflicts to declare.

## Supplementary Material

SC-012-D1SC00722J-s001

SC-012-D1SC00722J-s002

SC-012-D1SC00722J-s003

SC-012-D1SC00722J-s004

SC-012-D1SC00722J-s005
