# Supplementary material for: Simultaneously achieving high capacity storage and multilevel anti-counterfeiting using electrochromic and electrofluorochromic dual-functional AIE polymers
Source: Chem Sci. 2021 Apr 12;12(20):7058–65. doi: 10.1039/d1sc00722j (PMC8153231; doi:10.1039/d1sc00722j)
Supplement: SC-012-D1SC00722J-s001 [file SC-012-D1SC00722J-s001.pdf]

Electronic Supplementary Material (ESI) for Chemical Science.
This journal is © The Royal Society of Chemistry 2021

Electronic Supplementary Information

Simultaneously achieving high capacity storage and multilevel anti-counterfeiting using electrochromic and electrofluorochromic dual-functional AIE polymers

Lin Lu,[a] Kaojin Wang,[a] Haozhong Wu,[a] Anjun Qin[a,*] and Ben Zhong Tang[a, b]

[a] State Key Laboratory of Luminescent Materials and Device, Guangdong Provincial Key Laboratory of Luminescence from Molecular Aggregates, AIE Institute, Center for Aggregation-Induced Emission, South China University of Technology, Guangzhou 510640, China

[b] Shenzhen Institute of Aggregate Science and Technology, School of Science and Engineering, The Chinese University of Hong Kong, Shenzhen, Guangdong 518172, China

* Corresponding author email address: msqinaj@scut.edu.cn

Experimental section

Materials

Tetrahydrofuran (THF) and toluene were distilled from sodium benzophenone ketyl under dry nitrogen immediately prior to use. All other chemicals and reagents were purchased from commercial sources and used as received without further purification.

Instruments

^{1}H and ^{13}C NMR spectra were measured on a Bruker Avance 500 MHz NMR spectrometer using tetramethylsilane (TMS, δ = 0) as internal reference. High resolution mass spectra (HRMS) were performed on a GCT Premier CAB 048 mass spectrometer. The number- (M_n) and weight-average (M_w) molecular weights and polydispersity indices ($Đ = M_w/M_n$) of polymers were estimated by a Waters advanced polymer chromatography (APC) system, and THF was used as eluent at a flow rate of 0.5 mL/min. A set of monodispersed linear polystyrenes coving the M_w range of 10^3-10^7 g/mol were utilized as standards for molecular weight calibration. Thermogravimetric analysis (TGA) was performed on a TA TGA Q5000 under nitrogen atmosphere at a heating rate of 20 ^{o}C/min. Differential scanning calorimetry (DSC) analysis was carried out on a DSC Q1000 under nitrogen atmosphere at a heating rate of 10 ^{o}C/min. UV-vis absorption spectra were recorded with a Shimadzu UV-2600 spectrophotometer. Fluorescence spectra were recorded on a Horiba Fluoromax-4 spectrophotometer. Absolute photoluminescence quantum yields were measured using a Hamamatsu absolute PL quantum yield spectrometer C11347 Quantaurus QY. The electrochemical measurements were performed on CHI 660e electrochemical workstation. Cyclic voltammetry tests were conducted using polymer film/indium-tin oxide (ITO) as working electrode, a Ag/AgCl, KCl (sat.) as reference electrode, a platinum wire as auxiliary electrode, and 0.1 M tetrabutylammonium perchlorate (TBAP)/CH_3CN as supporting electrolyte.

Synthesis and characterization

Synthesis of 4-methoxy-*N*,*N*-bis[4-(4,4,5,5-tetramethyl-1,3,2-dioxaborolan-2-yl)phenyl]benzenamine (TPA-OMe). 4,4'-Dibromo-4''-methoxytriphenylamine (2.15 g, 5 mmol), bis(pinacolato)diboron (3.81 g, 15 mmol), potassium acetate (2.94 g, 30 mmol), and $Pd(OAc)_2$ (0.14 g, 0.6 mmol) were added into 100 mL two-neck flask under nitrogen atmosphere. Dry 1,4-dioxane (40 mL) was injected into the flask, and the mixture was stirred at 80 °C for 12 h. After reaction, the solvent was removed under reduced pressure. The solid was dissolved in DCM and washed with water and brine. Then the organic layer was dried over anhydrous Na_2SO_4. After filtration and solvent evaporation, the crude product was purified by column chromatography using petroleum ether (PE)/DCM (1:1 v/v) as eluent and a white solid (TPA-OMe) was obtained in 75% yield (1.97 g). ^{1}H NMR (500 MHz, CD_2Cl_2) δ 7.59 (d, 4H), 7.10-6.92 (m, 6H), 6.87 (d, 2H), 3.79 (s, 3H), 1.31 (s, 24H). ^{13}C NMR (125 MHz, CD_2Cl_2) δ 158.37, 151.53, 141.04, 136.97, 129.50, 122.89, 116.17, 84.85, 56.73, 25.95.

Synthesis of 1,2-di(4-bromophenyl)-1,2-diphenylethylene (TPE-Br). 4-Bromobenzophenone (2.59 g, 10 mmol), zinc powder (3.90 g, 60 mmol), and dry THF (60 mL) were added into 100 mL two-necked flask under nitrogen atmosphere. $TiCl_4$ (3.30 mL, 30 mmol) was injected into the flask at 0 °C, and the mixture was then refluxed for 24 h. The reaction was quenched by adding ammonium chloride solution. The organic layer was extracted with ethyl acetate (EA) and brine three times. Then the collected organic phase was dried over anhydrous Na_2SO_4. After filtration and solvent evaporation, this crude product was purified by column chromatography using PE/DCM (20:1 v/v) as an eluent and a white solid (TPE-Br) was obtained in 85% yield (2.07 g). ^{1}H NMR (500 MHz, CD_2Cl_2) δ 7.31-7.17 (m, 4H), 7.15-7.09 (m, 6H), 7.03-6.98 (m, 4H), 6.92-6.85 (m, 4H). ^{13}C NMR (125 MHz, CD_2Cl_2) δ 143.95, 143.55, 143.31, 142.86, 140.75, 133.30, 131.49, 131.18, 128.35, 128.11, 127.22, 126.99, 120.95.

Synthesis of 1,2-di(2-bromothiophene)-1,2-diphenylethylene (DTDPE-Br). 2-Benzoylthiophene (2.65 g, 10 mmol), zinc powder (3.90 g, 60 mmol), and dry THF (60 mL) were added into 100 mL two-necked flask under nitrogen atmosphere. $TiCl_4$ (3.30 mL, 30 mmol) was injected into the flask at 0 °C, and the mixture was then stirred at 40 °C for 24 h. The reaction was quenched by ammonium chloride solution. The organic layer was extracted with EA and brine three times. The collected organic phase was dried over anhydrous Na_2SO_4. After filtration and solvent evaporation, this crude product was purified by column chromatography using PE/DCM (20:1 v/v) as an eluent

and a light yellow solid (DPDPE-Br) was obtained in 20% yield (0.49 g). ^{1}H NMR (500 MHz, $CDCl_3$) δ 7.49-7.44 (m, 1.2H), 7.37-7.33 (m, 0.8H), 7.12-7.03 (m, 8H), 6.87 (d, 1.6H), 6.64 (d, 0.4H), 6.59 (d, 1.6H), 6.04 (d, 0.4H). ^{13}C NMR (125 MHz, $CDCl_3$) δ 146.74, 141.65, 140.20, 134.31, 132.52, 130.94, 130.80, 130.08, 130.01, 129.83, 129.43, 128.81, 128.64, 127.78, 127.28,114.70, 114.12. HRMS: *m*/*z* calcd for $C_{22}H_{14}Br_2S_2$: 501.8883 [M+]$^+$; found: 501.8909.

Synthesis of P(TPE-TPA). TPE-Br (97.6 mg, 0.2 mmol), TPA-OMe (105.5 mg, 0.2 mmol), a drop of Aliquat 336, and $Pd(PPh_3)_4$ (8.0 mg, 0.007 mmol) were added into 20 mL Schlenk tube under nitrogen atmosphere. Then K_2CO_3 aqueous solution (0.8 mL, 2.0 M) and toluene (4 mL) were added in one portion, and the mixture was reacted at 85 °C for 48 h. Bromobenzene (0.1 mL, 1.0 mmol) was added and the mixture further react for 12 h. Phenylboronic acid (242.0 mg, 2.0 mmol) was then added and allowed to react for another 12 h. The reaction mixture was cooled to room temperature and precipitated in methanol. The crude product was purified by Soxhlet extraction in methanol and acetone. Afterward, the polymer was dissolved in THF, precipitated in methanol, and a green solid was obtained in 60% yield (91.0 mg). M_w: 12 500, M_w/M_n: 1.48. ^{1}H NMR (500 MHz, CD_2Cl_2) δ 7.60-7.50 (m), 7.49-7.19 (m), 7.17-6.92 (m), 6.89-6.72 (m), 3.83-3.62 (m). ^{13}C NMR (125 MHz, CD_2Cl_2) δ 156.73, 147.34, 144.00, 142.43, 140.79, 140.30, 138.48, 133.88, 131.82, 131.53, 131.09, 128.86, 128.26, 127.20, 126.58, 125.65, 122.98, 114.93, 55.55.

Synthesis of P(DTDPE-TPA). DTDPE-Br (100.0 mg, 0.2 mmol), TPA-OMe (105.5 mg, 0.2 mmol), a drop of Aliquat 336, and $Pd(PPh_3)_4$ (8.0 mg, 0.007 mmol) were added into 20 mL Schlenk tube under nitrogen. Then K_2CO_3 aqueous solution (0.8 mL, 2.0 M) and toluene (4.0 mL) were added in one portion, and the mixture was heated to 85 °C for 48 h. Bromobenzene (0.1 mL, 1.0 mmol) was added and left to react for additional 12 h. Phenylboronic acid (242.0 mg, 2.0 mmol) was added and allowed to react for another 12 h. The reaction mixture was cooled to room temperature and precipitated in methanol. The crude polymer was purified by Soxhlet extraction in methanol and acetone. After purification, the polymer was dissolved in THF, precipitated in methanol, and an orange solid was obtained in 65% yield (100.0 mg). M_w: 14 400, M_w/M_n: 1.56. ^{1}H NMR (500 MHz, $CDCl_3$) δ 7.59 (d), 7.47-7.37 (m), 7.37-7.29 (m), 7.22-7.07 (m), 7.06-6.97 (m), 6.90 (d), 6.80 (s), 6.25 (s), 3.77 (s). ^{13}C NMR (125 MHz, $CDCl_3$) δ 156.49, 146.86, 144.87, 144.51, 142.70, 141.78, 139.87, 134.36, 132.69, 131.05, 129.01, 128.74, 127.73, 127.62, 127.38, 127.15, 126.68, 126.30, 126.21, 122.76, 121.78, 121.12 , 114.83, 55.47.

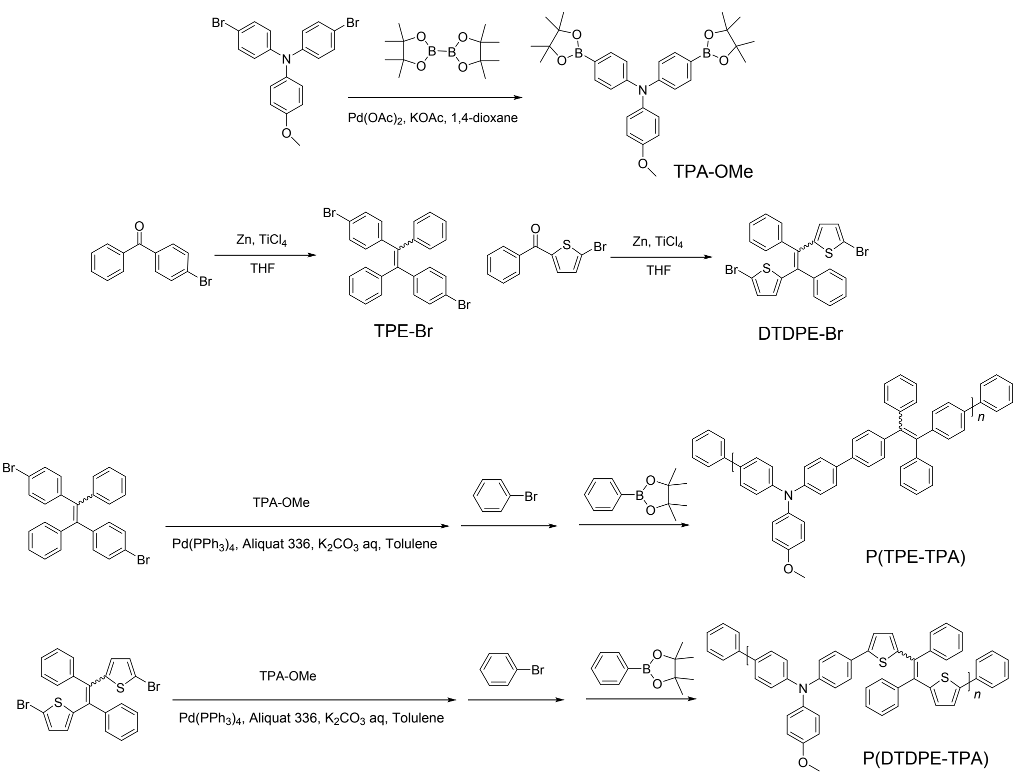

Scheme S1. Synthetic routes to P(TPE-TPA) and P(DTDPE-TPA)

Synthesis of TPE-NB. TPE-OH (0.816 g, 2.2 mmol), exo-5-norbornene carboxylic acid (0.27 g, 2 mmol), 4-dimethylaminopyridine (48 mg, 0.4 mmol), and 30 mL anhydrous DCM were added in a 50 mL round-bottom flask. After stirring at room temperature for 0.5 h, *N*,*N*'-dicyclohexylcarbodiimide (0.41 g, 4 mmol) was added to the system under an ice bath. The mixture was allowed to react overnight at room temperature. Afterward, the insoluble salt was filtered, and the filtrate was evaporated to get the crude product. Then the crude product was purified by column chromatography using PE/DCM (1:1 v/v) as eluent, and a yellowish-white solid (TPE-NB) was obtained in 70% yield (0.73 g). ^{1}H NMR (400 MHz, $CDCl_3$) δ 7.08 (s, 3H), 7.01 (d, 4H), 6.93 (d, 4H), 6.84 (d, 2H), 6.64 (d, 4H), 6.17 (d, 2H), 3.82-3.65 (m, 6H), 3.49 (d, 1H), 3.18 (s, 1H), 2.97 (s, 1H), 2.43 (d, 1H), 2.06-1.96 (m, 1H), 1.64-1.49 (m, 2H). ^{13}C NMR (100 MHz, $CDCl_3$) δ 174.74, 158.16, 149.08, 144.07, 141.69, 140.41, 138.21, 136.20, 135.69, 132.46, 132.27, 131.95, 131.44, 127.74, 126.19, 120.70, 113.14, 55.11, 46.78, 46.42, 43.37, 41.76, 30.57.

Synthesis of P(TPE-NB). Under nitrogen atmosphere, TPE-NB (105.64 mg, 0.2 mmol) and Grubbs third generation catalyst (0.88 mg, 0.001 mmol) were added into 10 mL Schlenk tube. Then, 2 mL anhydrous DCM was quickly injected into the tube, and the reaction was stirred for 2 h at room temperature. After the reaction, 0.1 mL vinyl

ether was added to quench the reaction. After stirring for 1 h at room temperature, the reaction mixture was diluted with DCM and precipitated in methanol. The procedures of dissolution and precipitation were repeated three times. A yellowish-white solid was obtained in 60% yield (63.0 mg). ^{1}H NMR (500 MHz, $CDCl_3$) δ 7.16-6.86 (m), 6.83-6.67 (m), 6.65-6.50 (m), 5.57-5.15 (m), 3.76-3.56 (m), 3.14 (d), 2.80-2.57 (m), 2.26-1.87 (m), 1.79-1.54 (m), 1.23 (d). ^{13}C NMR (125 MHz, $CDCl_3$) δ 173.74, 158.13, 149.01, 143.98, 141.67, 140.32, 138.22, 136.07, 132.56, 131.40, 127.71, 120.68, 112.98, 55.05, 50.29, 41.98, 36.98. M_w: 180 100, M_w/M_n: 1.85.

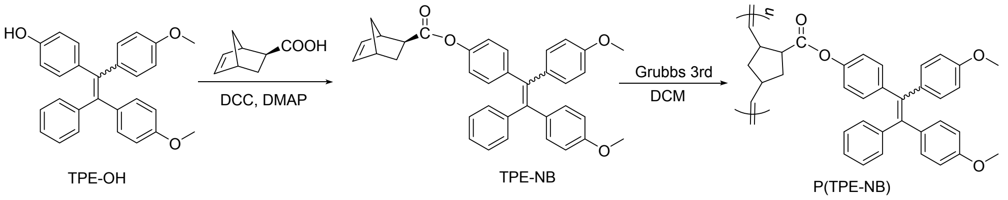

Scheme S2. Synthetic routes to P(TPE-NB)

The details of device fabrication. Herein, the device with "X" and "Y" patterns was taken as an example: The device configuration is ITO glass/polymer/gel electrolyte/ITO glass. First, the PVC sticker (thickness: 0.5 mm) was designed into hollowed-out "X" and "Y" mold (the area of the pattern is 1.5 × 1.5 cm), and then pasted onto the ITO glass (4.5×5.5 cm). Then, the "Y" area was blocked with a plastic sheet, and P(DTDPE-TPA) chloroform solution (5 mg/mL) was sprayed for three times to the area of "X" by a spray gun. Following the same procedure, P(TPE-TPA) was sprayed to the area of "Y". After removing the PVC mold, the gel electrolyte is covered on the polymer-coated ITO glass, and another piece of ITO glass is put on the electrolyte. To prevent leakage of the gel electrolyte, the edges of the assembled device are sealed with tape. Finally, the "X" and "Y" information display device was successfully constructed, and the voltage generator was used to exert different voltage values on the device.

Using the similar procedure, the numerical devices were fabricated. The length and width of each small rectangle are 1.3 cm and 0.3 cm respectively. The size of ITO glass is 4.8×4.8 cm. For multilevel anti-counterfeiting device, the individual squares are 0.5 cm in size and the middle spaces are 0.1 cm.

The detailed synthesis procedures of gel electrolyte. Tetrabutylammonium perchlorate (TBAP) was dissolved in mixed solvents of anhydrous acetonitrile (ACN) and propylene carbonate (PC). The above solution was stirred for 10 min at 70 ºC. Then, polymethyl methacrylate (PMMA) was added, and the system was stirred vigorously for additional 1 h. Finally, a uniform gel electrolyte system was obtained, where CAN/PC/PMMA/TBAP = 70:20:7:3 (wt%).

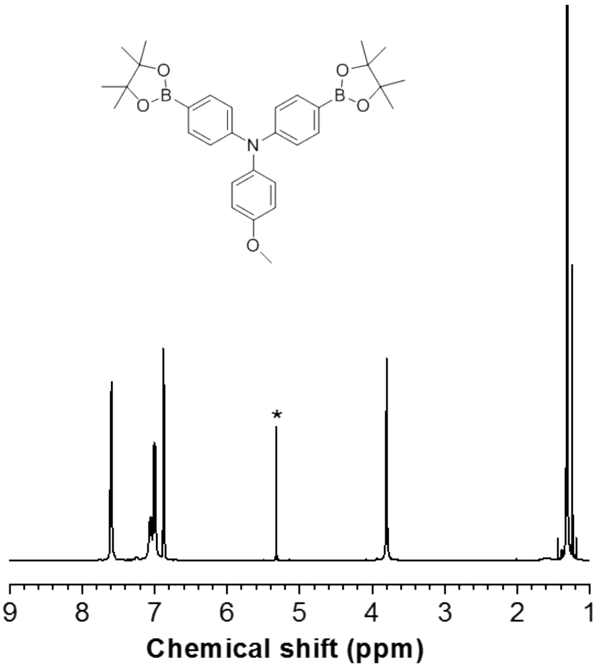

Figure S1. ^{1}H NMR spectrum of TPA-OMe in CD_2Cl_2. The solvent peak is marked with asterisk.

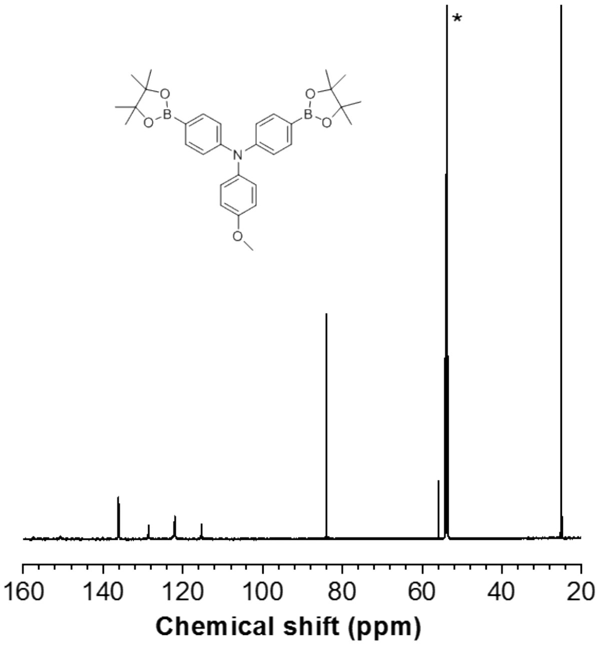

Figure S2. ^{13}C NMR spectrum of TPA-OMe in CD_2Cl_2. The solvent peak is marked with asterisk.

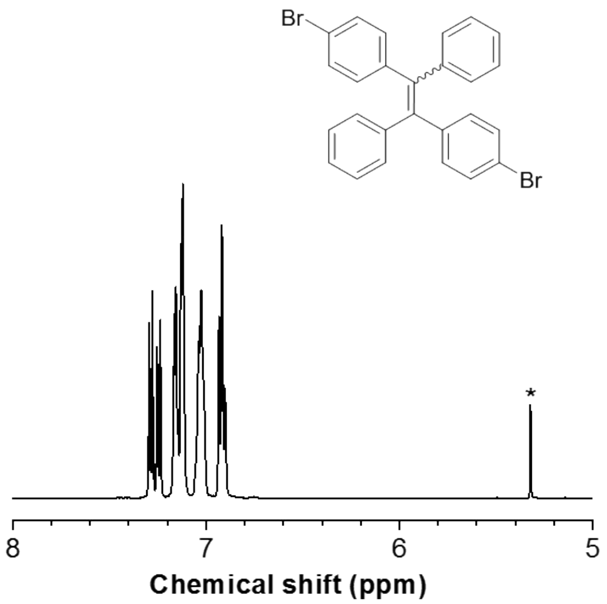

Figure S3. ^{1}H NMR spectrum of TPE-Br in CD_2Cl_2. The solvent peak is marked with asterisk.

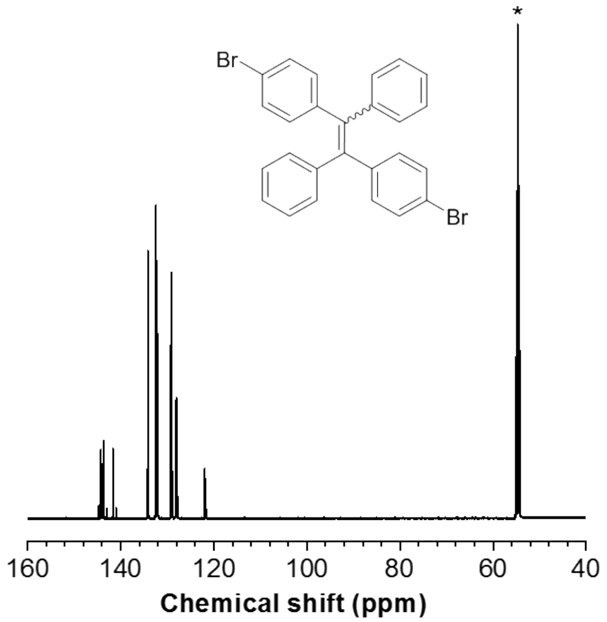

Figure S4. ^{13}C NMR spectrum of TPE-Br in CD_2Cl_2. The solvent peak is marked with asterisk.

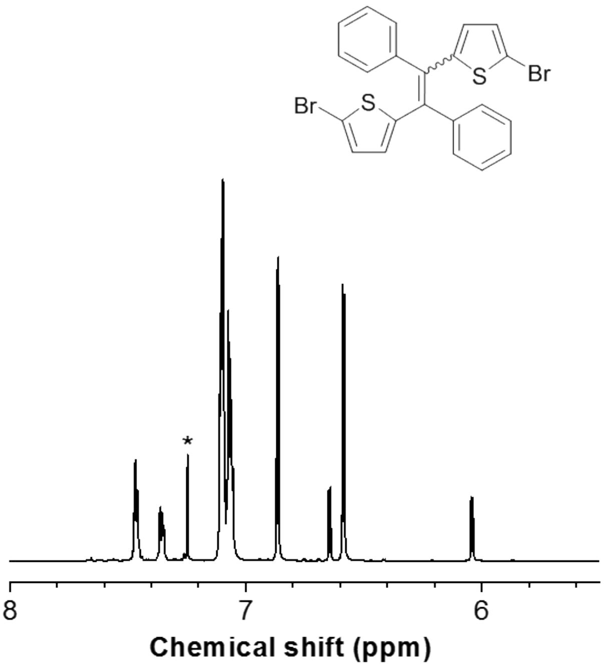

Figure S5. ^1H NMR spectrum of DPDPE-Br in $CDCl_3$. The solvent peak is marked with asterisk.

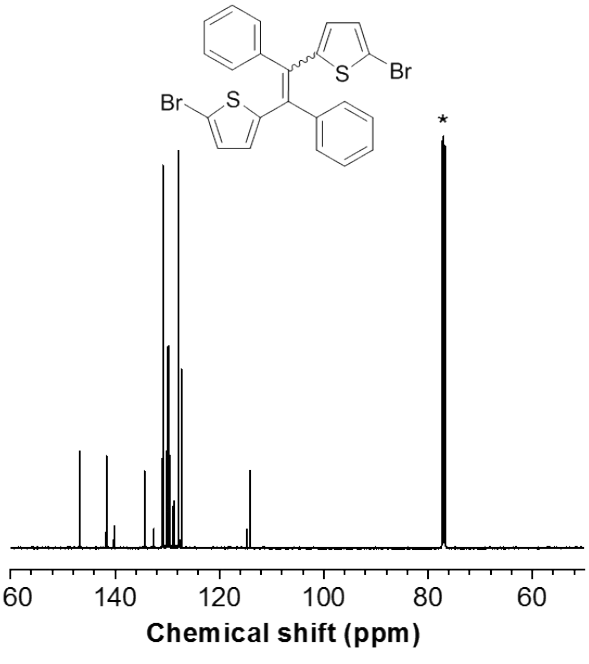

Figure S6. ^{13}C NMR spectrum of DPDPE-Br in $CDCl_3$. The solvent peak is marked with asterisk.

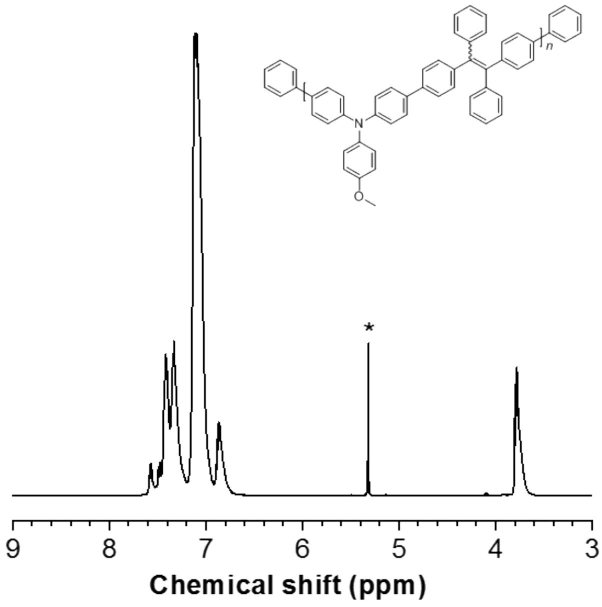

Figure S7. ^{1}H NMR spectrum of P(TPE-TPA) in CD_2Cl_2. The solvent peak is marked with asterisk.

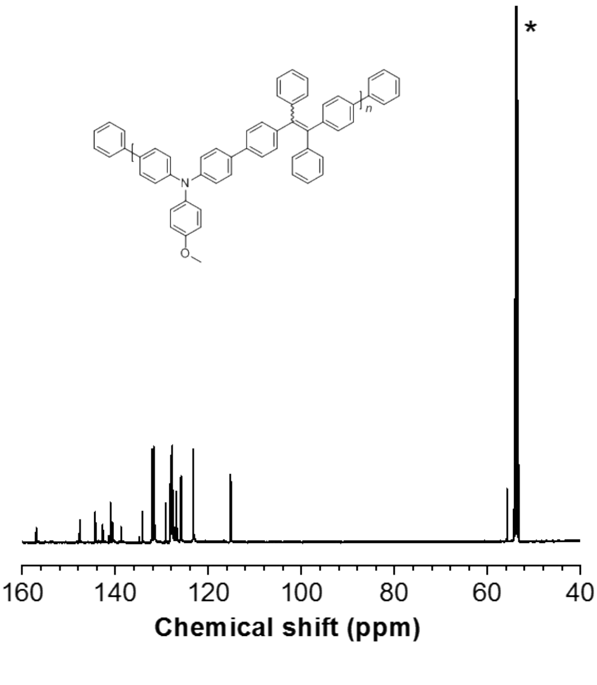

Figure S8. ^{13}C NMR spectrum of P(TPE-TPA) in CD_2Cl_2. The solvent peak is marked with asterisk.

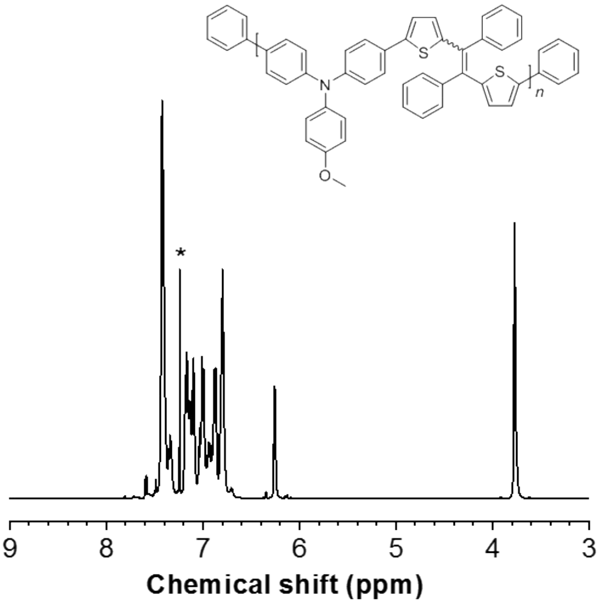

Figure S9. ^{1}H NMR spectrum of P(DTDPE-TPA) in $CDCl_3$. The solvent peak is marked with asterisk.

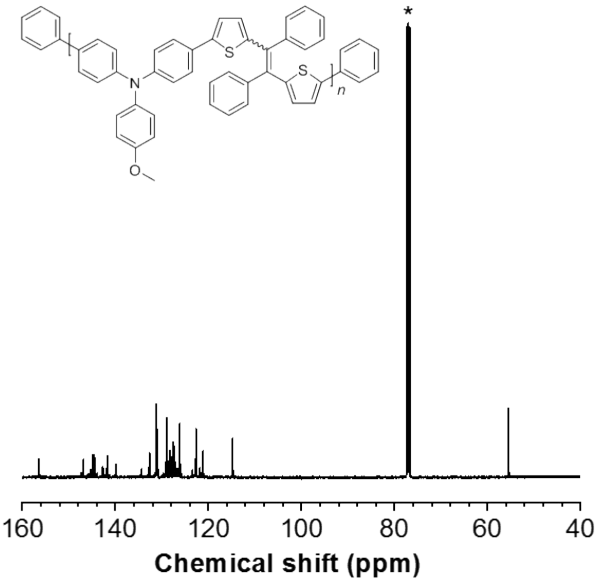

Figure S10. ^{13}C NMR spectrum of P(DTDPE-TPA) in $CDCl_3$. The solvent peak is marked with asterisk.

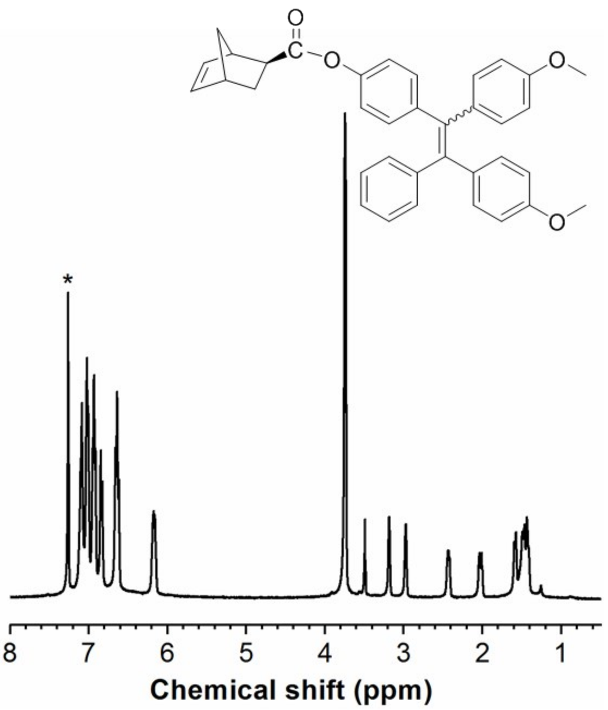

Figure S11. ^{1}H NMR spectrum of TPE-NB in $CDCl_3$. The solvent peak is marked with asterisk.

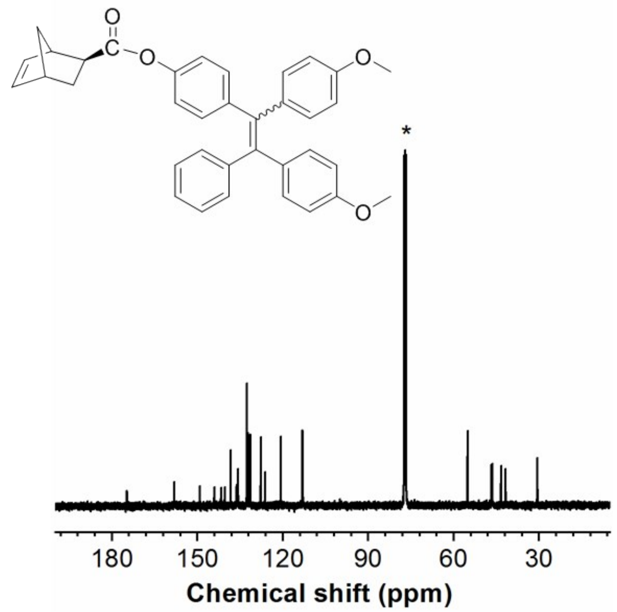

Figure S12. ^{13}C NMR spectrum of TPE-NB in $CDCl_3$. The solvent peak is marked with asterisk.

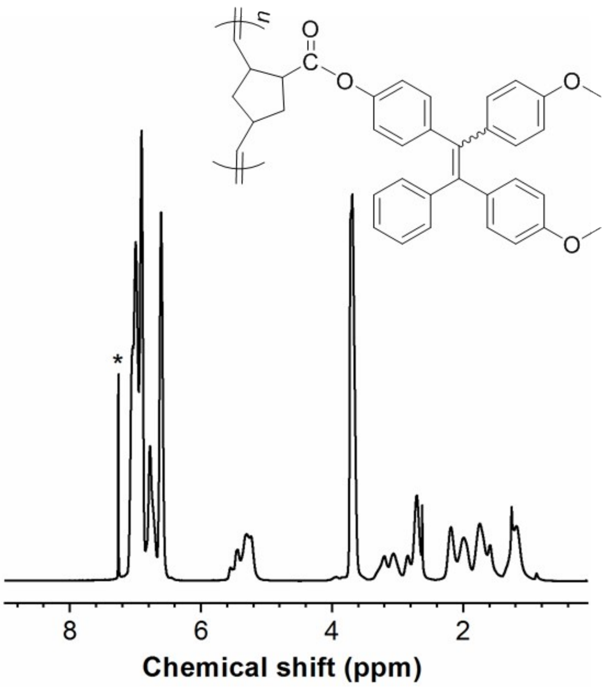

Figure S13. ^1H NMR spectrum of P(TPE-NB) in $CDCl_3$. The solvent peak is marked with asterisk.

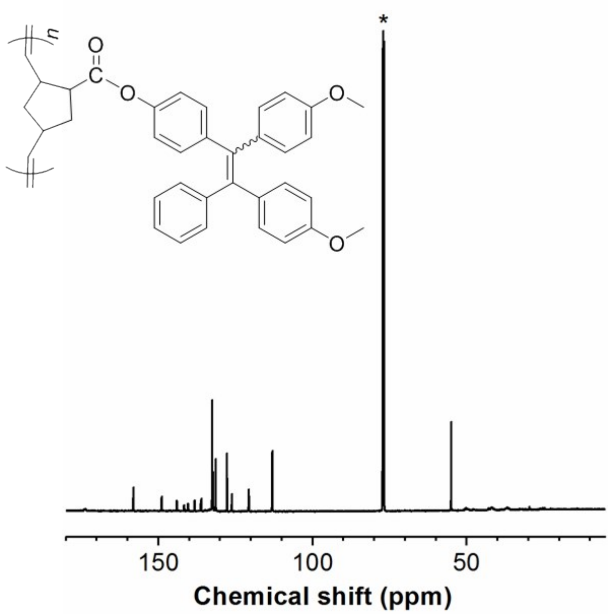

Figure S14. ^{13}C NMR spectrum of P(TPE-NB) in $CDCl_3$. The solvent peak is marked with asterisk.

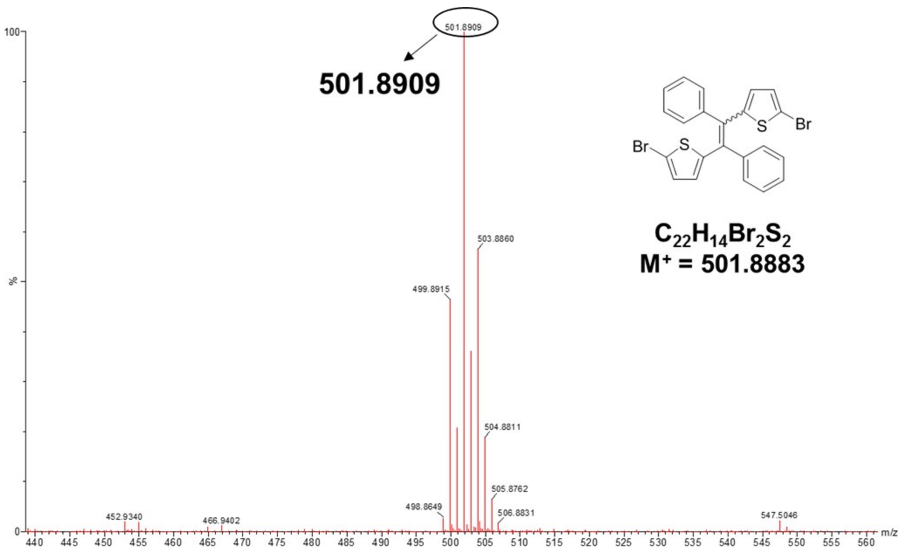

Figure S15. HRMS spectrum of DTDPE-Br.

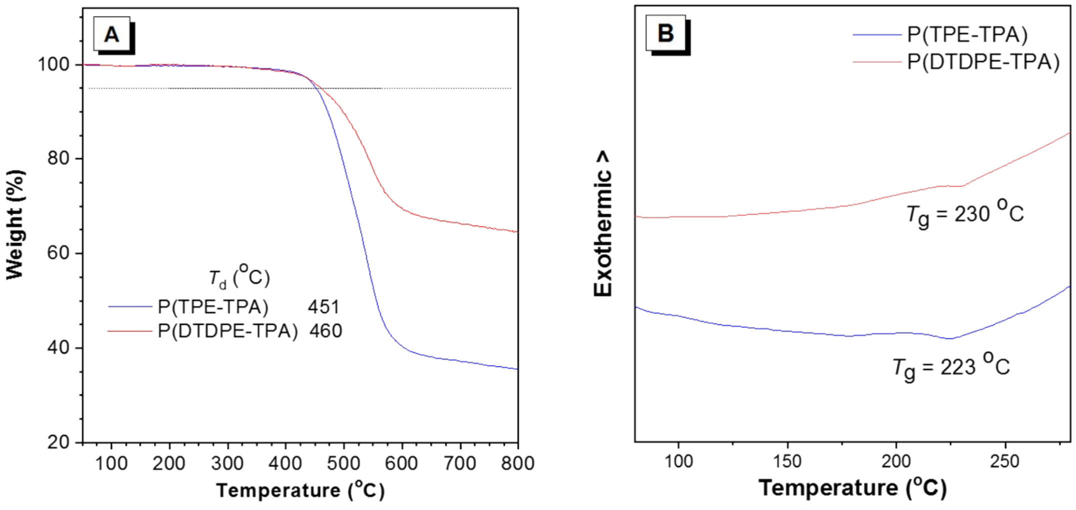

Figure S16. (A) TGA and (B) DSC thermograms of P(TPE-TPA) and P(DTDPE-TPA) recorded under nitrogen at a heating rate of (A) 20 °C/min and (B) 10 °C/min, respectively.

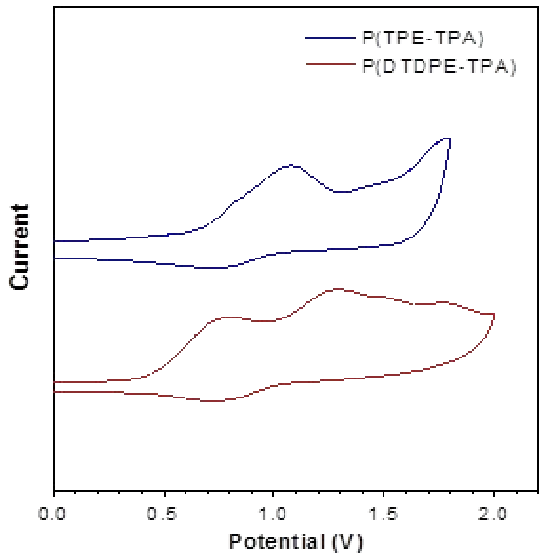

Figure S17. CV curves of P(TPE-TPA) and P(DTDPE-TPA) films immobilized on ITO-coated glass slides in 0.1 M TBAP/CH_3CN solution with a scan rate of 100 mV/s.

Table S1. Optical and electrochemical properties of P(TPE-TPA) and P(DTDPE-TPA).

Polymer	$\lambda_{abs}{}^{a}$ (nm)	$\lambda_{em}{}^{b}$ (nm)	Φ^{c} (%) in soln	Φ^{c} (%) in film	$E_{onset}{}^{d}$ (V)
P(TPE-TPA)	372	513	2.2	52.9	0.62
P(DTDPE-TPA)	441	585	0.7	15.6	0.43

[a] Maximum absorption wavelength, concentration: 10 M. [b] Maximum emission wavelength, soln: THF solution. [c] Absolute fluorescence quantum efficiency. [d] Onset oxidation potential of the polymers CV curve.

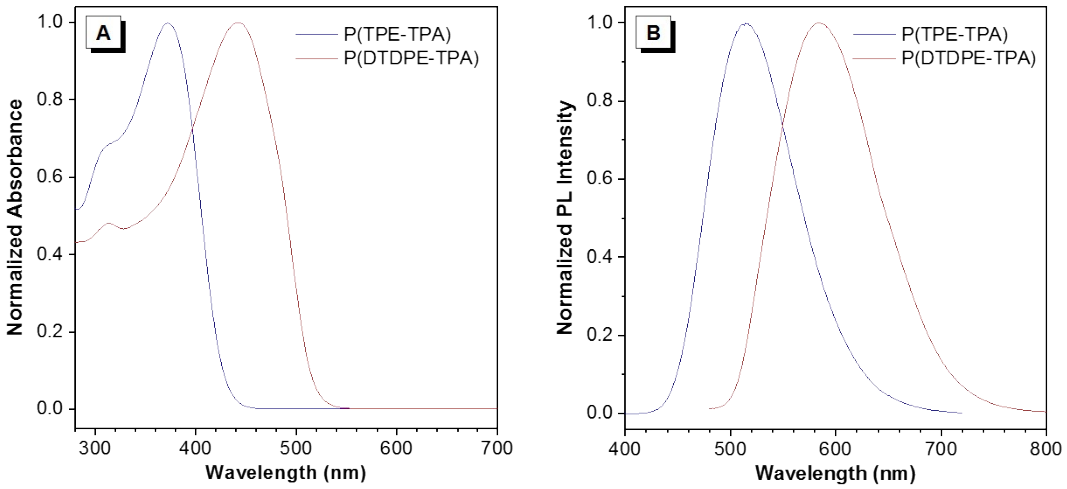

Figure S18. (A) UV and (B) PL spectra of P(TPE-TPA) and P(DTDPE-TPA) in the THF solutions. Concentration: 10 μM.

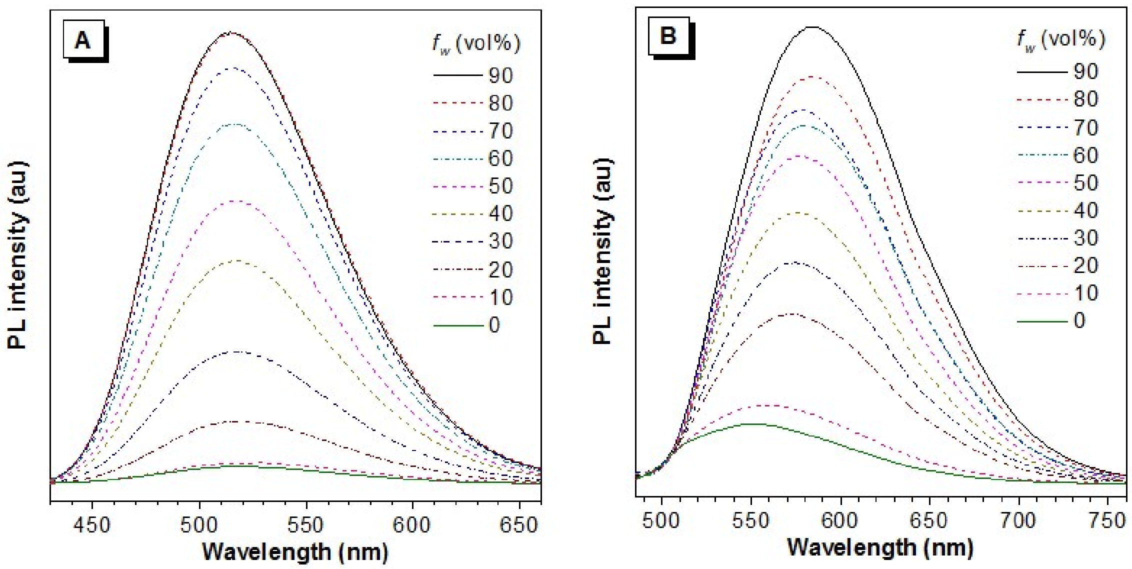

Figure S19. PL spectra of (A) P(TPE-TPA) and (B) P(DTDPE-TPA) in THF/water mixtures with different water fractions (f_w). Concentration: 10 μM; λ_{em}: 370 nm for P(TPE-TPA); 440 nm for P(DTDPE-TPA).

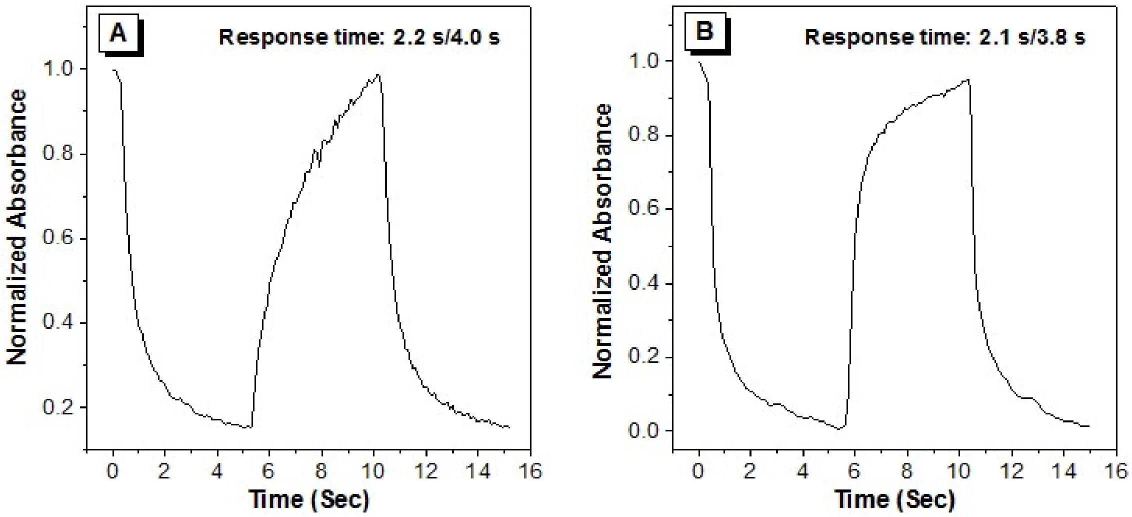

Figure S20. (A) Absorbance switching time of P(TPE-TPA) film electrode between 0 and 1.2 V monitored at 373 nm; (B) Absorbance switching time of P(DTDPE-TPA) film electrode between 0 and 1.0 V monitored at 435 nm.

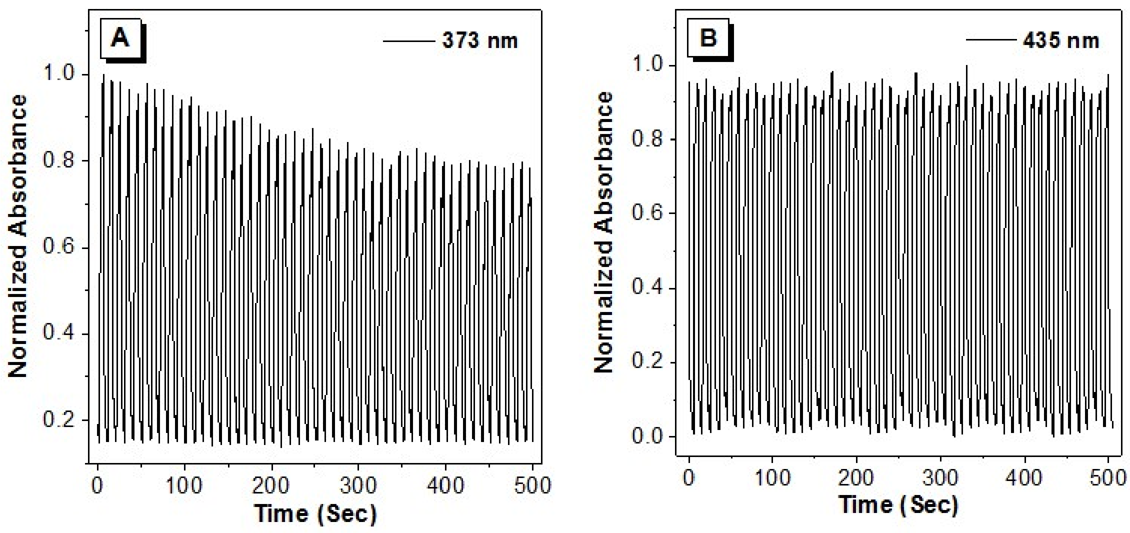

Figure S21. (A) Repeated absorbance changes of P(TPE-TPA) film electrode monitored at 373 nm under applied potentials between 0 and 1.2 V with duration time set as 10 s; (B) Repeated absorbance changes of P(DTDPE-TPA) film electrode monitored at 435 nm under applied potentials between 0 and 1.0 V with duration time set as 10 s.

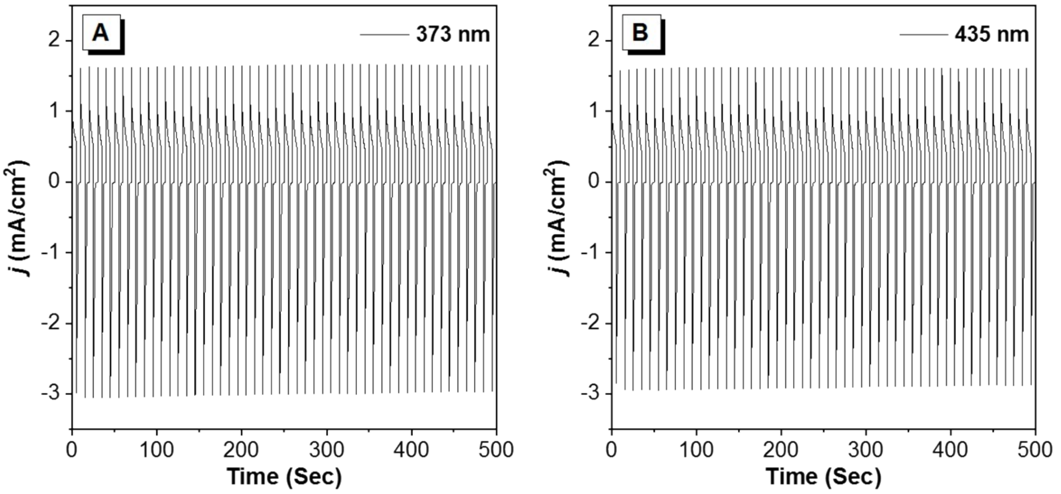

Figure S22. Chronoamperometry curves of (A) P(TPE-TPA) and (B) P(DTDPE-TPA) film electrodes (coated area: 3 cm × 0.7 cm) with duration time set as 10 s.

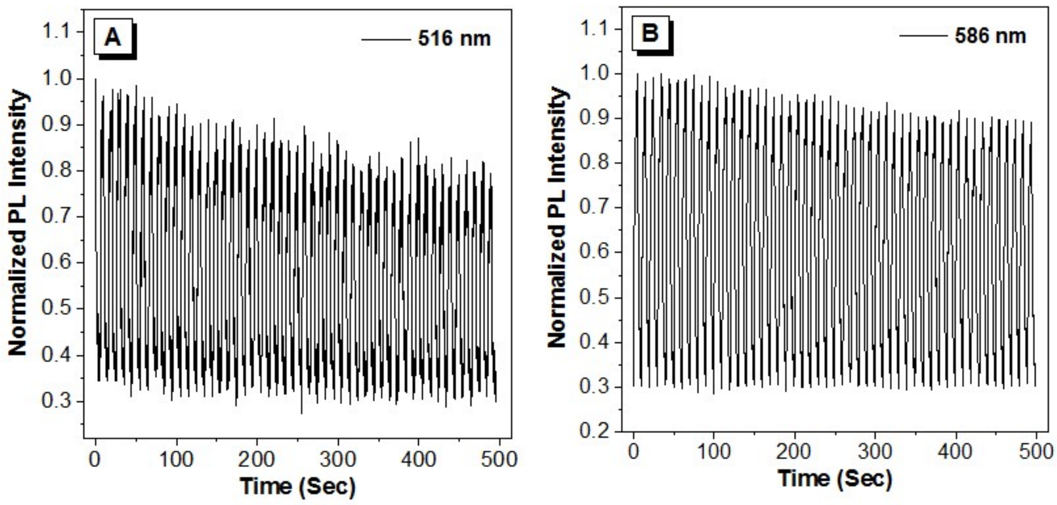

Figure S23. (A) PL switching of P(TPE-TPA) film electrode monitored at 516 nm under applied potentials between 0 and 1.0 V with duration time set as 10 s; (B) PL switching of P(DTDPE-TPA) film electrode monitored at 586 nm under applied potentials between 0 and 0.7 V with duration time set as 10 s.

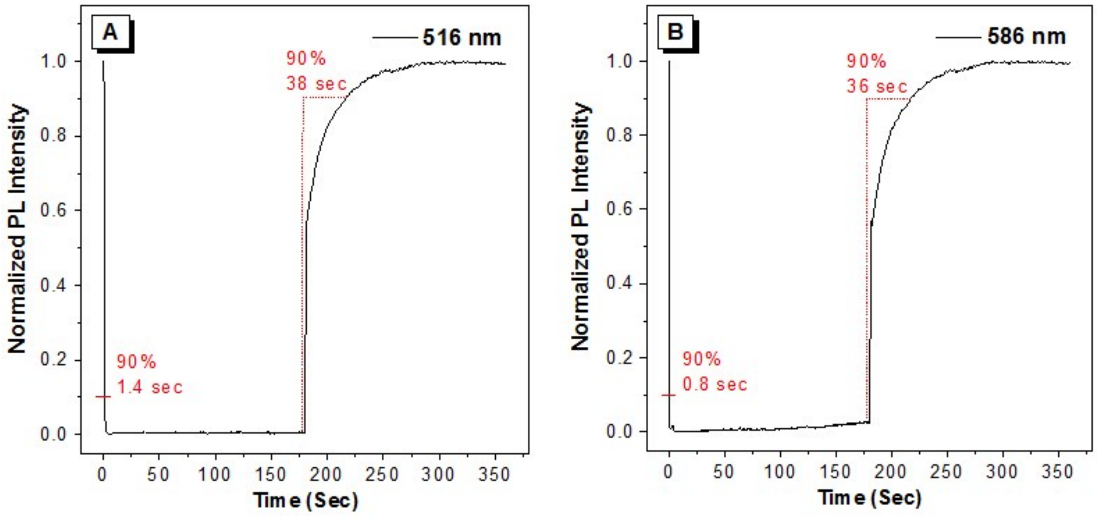

Figure S24. (A) Estimation of PL switching time of P(TPE-TPA) film electrode between 0 and 1.0 V monitored at 516 nm; (B) Estimation of PL switching time of P(DTDPE-TPA) film electrode between 0 and 0.7 V monitored at 586 nm.

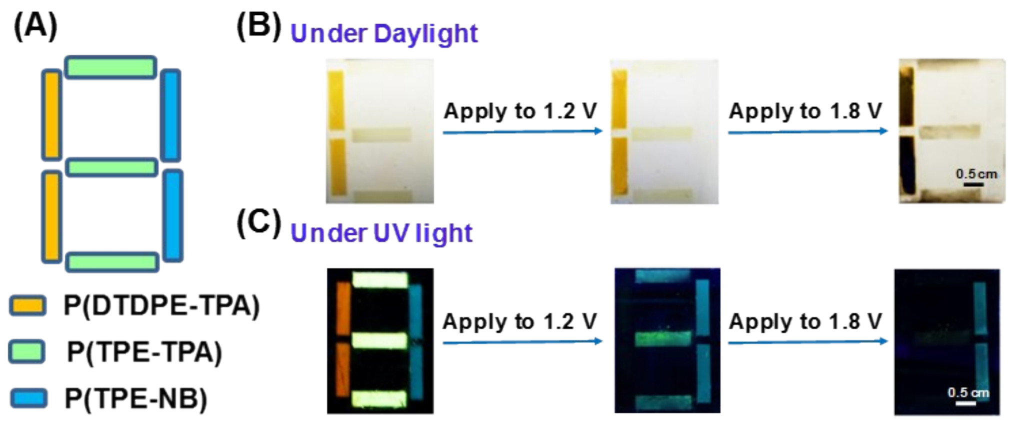

Figure S25. (A) Patterns designed using P(TPE-TPA), P(DTDPE-TPA) and P(TPE-NB). The numerical device based on three polymers with different voltages under daylight (B) or UV light (C). The size of the ITO substrate is 4.8 × 4.8 cm.

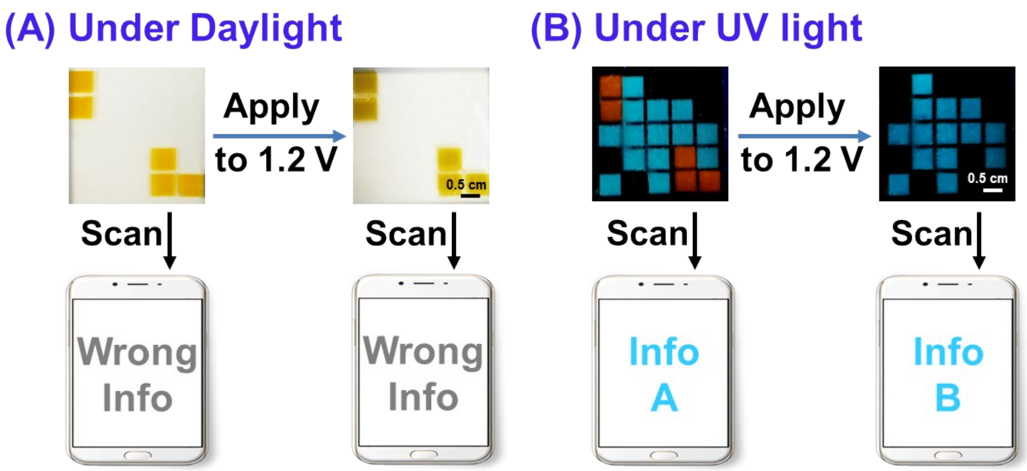

Figure S26. The color code device based on P(TPE-TPA) and P(DTDPE-TPA) with different voltages under daylight (A) and UV light (B). The size of the ITO substrate is 4.8 × 4.8 cm.
